# Experiences of antipsychotic use in patients with early psychosis: a two-year follow-up study

**DOI:** 10.1186/s12888-017-1425-9

**Published:** 2017-08-22

**Authors:** Rafal A. H. Yeisen, Jone Bjornestad, Inge Joa, Jan Olav Johannessen, Stein Opjordsmoen

**Affiliations:** 1 Centre for Clinical Psychosis Research, Division of Psychiatry, Stavanger University Hospital, Stavanger, Norway; 2 Stavanger Hospital pharmacy, Western Norway Pharmaceutical Trust, Stavanger, Norway; 3 Network for medical sciences, Faculty of Social Sciences, University of Stavanger, Stavanger, Norway; 4 Division of Mental Health and Addiction, Oslo University Hospital, Oslo, Norway; 5 Institute of Clinical Medicine, University of Oslo, Oslo, Norway

## Abstract

**Background:**

Non-adherence is a major public health problem despite treatment advances. Poor drug adherence in patients with psychosis is associated with more frequent relapse, re-hospitalization, increased consumption of health services and poor outcomes on a variety of measures. Adherence rate in patients with first episode psychosis have been found to vary from 40 to 60%. However, most previous studies have addressed the consequences of non-adherence rather than its potential causes.

The purpose of this study was, therefore, to investigate experiential factors which may affect adherence to medication in adults with psychotic disorders, during the 24-month period after the onset of treatment.

**Methods:**

Twenty first episode patients (7 male, 13 female) were included in our qualitative sub-study from the ongoing TIPS2 (Early Intervention in Psychosis study). Each person participated in semi-structured interviews at 2-year follow-up. All had used antipsychotics, with some still using them. Data were analyzed within an interpretative-phenomenological framework using an established meaning condensation procedure.

**Results:**

The textual analysis revealed four main themes that affected adherence largely: 1) Positive experiences of admission, 2) Sufficient timely information, 3) Shared decision-making and 4) Changed attitudes to antipsychotics due to their beneficial effects and improved insight into illness.

**Conclusion:**

Patients reported several factors to have a prominent impact on adherence to their antipsychotics. The patients do not independently choose to jeopardize their medication regime. Health care staff play an important role in maintaining good adherence by being empathetic and supportive in the admission phase, giving tailored information according to patients’ condition and involving patients when making treatment decisions.

## Background

Antipsychotic medications have proven to be efficacious in the acute stage and short-term treatment of psychosis. Most studies have found missing drug regimens and poor adherence to be associated with increased risk for relapse and re-hospitalization, along with increased consumption of health services, poorer quality of life and worse prognosis as measured by rates of remission and long-term recovery [1–5]. Long-term use of antipsychotics have been associated with brain volume reduction and supersensitivity of the dopamine D2 receptor [6]. However, no clinical trial have so far answered the question of whether antipsychotics adversely affect the long-term course of the illness. Most patients with schizophrenia seem to improve on antipsychotics, but for the individual patient the beneficial effects have to be balanced against potential side-effects [7–9].

Rates of adherence to antipsychotics during the first-episode psychosis (FEP) vary across studies, but overall findings indicate that approximately 40–60% of patients fully adhere [10–13]. Adherence is commonly considered “good”, or patients are described as “adherent”, if they use at least 70–80% of their prescribed medication [14, 15]. Non-adherence is still considered a significant public health problem despite treatment advances [16, 17].

Shared decision-making has become a stated priority in medical treatment in an attempt to increase adherence, subjective empowerment and reduced use of compulsory treatment [18, 19]. In somatic healthcare, shared decision making has already been found to be beneficial to adherence [20]. However, few studies have investigated the effects of shared decision-making in mental healthcare settings [21]. Although clinical guidelines recommend professionals to take into account patient opinions when choosing antipsychotic medication (AP) [22], these guidelines are not well implemented [23].

For a patient to accept the idea of long-term drug treatment involves a series of complex decisions with many potential pitfalls [24]. Most studies investigating aspects of non-adherence have focused on consequences rather than its causes. However some studies have studied different reasons of non-adherence which could be either medication-related like adverse effects and lack of efficacy, patient’s related linked to personal choices or disease-related factors [25, 26]. Increasing the knowledge of patients’ subjective experiences with antipsychotics would broaden our horizon to understand adherence issue better.

## Aim of the study

To investigate the subjective experiences and attitudes of patients with first episode psychosis with regard to adherence to antipsychotic medication during the first two years following of treatment.

## Methods

We used a thematic analytic approach [27, 28] within an interpretative-phenomenological framework [29, 30]. In this study, the interpretative element implies that data were generated from a reflexive dialogue between participants and researcher throughout the interview. The phenomenological element entails that significant knowledge was collected from individuals with lived experience of mental health problems, and that the central aim was to discover and interpret the meaning of such experiences within their broader contexts [31]. This study was approved by the Regional Ethics Committee in Norway (2015/72/REK vest). Written informed consent was obtained.

### Sample and recruitment

Participants were recruited from the ongoing TIPS-2 study (early intervention and treatment of psychosis), a naturalistic follow-along FEP study based in South-Rogaland, Norway, which began inclusion in 2002. Detailed descriptions of the inclusion criteria and methods have been published elsewhere [32, 33]. Individuals included in the TIPS-2 study met the following criteria: living in the catchment area (Rogaland county); age 15–65 years; meeting the DSM-IV criteria (as measured by The Structured Clinical Interview (SCID) for the DSM-IV Axis 1 Disorders) [34]; Being actively psychotic, was defined; as measured by a Positive and Negative Syndrome Scales (PANSS) score of 4 or more on at least one of positive subscale items 1 (delusions), 3 (hallucinatory behaviour), 5 (grandiosity), or 6 (suspiciousness/persecution) or general subscale item 9 (unusual thought content) for at least 7 days, being actively psychotic, as measured by a Positive and Negative Syndrome Scales (PANSS) score of 4 or more on at least one of positive subscale items 1 (delusions), 3 (hallucinatory behaviour), 5 (grandiosity), or 6 (suspiciousness/persecution) or general subscale item 9 (unusual thought content) for at least 7 days [35, 36]; not previously receiving adequate treatment of psychosis, was defined as “the start of structured treatment with antipsychotic medications (defined as antipsychotic medication of > 3,5 haloperidol equivalents for > 12 weeks or the start of hospitalization in highly staffed psychiatric wards organized to manage disturbing psychotic symptoms, not having received previous adequate treatment for psychosis or until remission of the psychotic symptoms)” [36]; no neurological or endocrine disorders related to the psychosis; understand and speaking a Scandinavian language; IQ < 70; and being able and willing to give informed consent to participate. The service-users agreed to baseline assessment and follow-up after 3 months, and 1, 2 and 5 years.

In this sub-study, participants were recruited consecutively when attending their 2-year follow-up sessions (calculated from inclusion date in the TIPS-2 study). Based on these assessments, only participants who had used antipsychotic medication were included. Twenty-six eligible candidates were contacted. Out of these five participants refused study participation and we were unable to obtain to consent from a sixth person. Sample size was decided on the basis of stability of findings, reviewed after 14 and 17 participants [37]. We stopped recruiting after including 20 participants, as we considered the last two interviews to not contribute substantially new information.

### Measures

SCID was used for diagnostic purposes. Psychosis symptoms were measured with PANSS. The Lehman Quality of Life Interview—brief version (L-QoLI) [38] was used to evaluate objective and subjective measures related to quality of life [39]. In our study, patients were assessed, by patient self-report, as to adhere to their medication if they followed their physician’s recommendations for at least 6 months prior to the 2-year follow-up, although adherence prior to this might have been irregular.

### Interviews

Interviews were conducted between June 2015 and January 2016. A semi-structured interview guide [40] was developed based on a collaboration between one recovered service-user counselor and the researchers, as well as literature regarding antipsychotic medication and adherence. The interview guide provided a list of key questions covering pre-defined themes such as; present and previous medication use; perceived medication effects; involvement and collaboration regarding use; information; safeguarding by professionals; adherence. Each theme was introduced with an open-ended question, for example, “Can you tell me about your experiences using antipsychotic medications?” The questions were followed-up depending on how much the participant elaborated. We tried to encourage participants to relate their experiences, asking questions such as, “You say that antipsychotics were particularly helpful in the acute phase, can you add some details on this please?” To capture topics not adequately covered by the interview, participants were invited at the end of each session to provide any information, which had not yet been elicited.

Pilot interviews were conducted with three FEP patients. The first author conducted all interviews, 18 of these at Stavanger University hospital, and the remaining two in participants’ homes. Interviews were audiotaped and transcribed verbatim for the purpose of analysis. The mean time and range of each interview: Mean: 51 min (range 36–74 min).

### Analysis

With a particular focus on experiences concerning antipsychotic adherence, semantic analysis employed a meaning condensation procedure [28] involving the six-steps presented in Table 1 [41]. To strengthen the credibility of the study, four researchers conducted the six-step procedure independently. Further, during five collaborative meetings this group of researchers compared their interpretations, agreed on themes with accompanying quotes, and validated the findings by consensus decision [37]. In the collaborative meetings we had a particular focus on steps four to six presented in Table 1. To overcome possible disagreement in the analytic process we agreed on the following decision rules in the preparatory phases of the study: 1) Resolve minor disagreement utilizing the principle of parsimoniousness (i.e. Occam’s razor: when two competing theories make exactly the same predictions, the simpler one is the more likely). 2) To resolve major disagreement we applied i) an inductive principle using the raw data as a compass, aiming to select the descriptions most closely reflecting the experience of the phenomena at issue. ii) Further we applied the principle of the best argument as described above.

**Table 1:** Steps of Text Condensation

1.	Becoming familiar with the data through thorough reading and re-reading of the transcribed interviews. Further, interviews were analyzed paragraph by paragraph to form a main impression of the experiences of the participants and to identify meaning units. A meaning unit is a basic text section that encapsulates one aspect of meaning as it relates to the experience of the informant and is identified by a spontaneous shift in the meaning of the text [41].
2.	Generating initial codes. A code consists of a coherent set of ‘meaning units’. These were further divided and sub-divided into groups.
3.	Searching for and developing candidate themes and sub-themes. A this stage a theme was defined as a verbalization capturing a set of important codes of the data in relation to the research question, representing a patterned response in the data set. Remaining themes were set aside at this phase in a separate category for the purpose of being further analyzed and incorporated when appropriate.
4.	Reviewing themes to develop a coherent thematic map and considering the validity of individual themes in relation to the data set. This is a process of checking that the suggested themes, on a semantic level, actually reflect the raw data.
5.	Defining and naming themes: Further refining and defining themes, identifying the essence of themes, identifying subthemes and summarizing the contents of the main themes into what each researcher considered to best represent participants’ experiences. When our refinements no longer added substantially to the themes, the analytic process was closed.
6.	To determine the relevance of a particular theme we both counted the frequency of the relevant meaning units combined with our interpretation of how central the theme was perceived to the recovery process.

## Results

Demographic and clinical characteristics of participants at baseline and after two-year follow-up are presented in Table 2. Fourteen (70%) of the included patients had no earlier psychiatric treatment history prior to the present episode.. Four patients had 1 earlier hospital stay, two patients had 2 stays and one patients had 4 hospital stays before first treatment for psychosis. (Mean length of these stays was 1.78 weeks). Most common earlier diagnosis was mild depression.

**Table 2:** Demographic and clinical characteristics of participants

	Baseline	2 year follow up
Gender		
Male (n)	7	
Female (n)	13	
Mean age (range)	24.6 (16–40)	
Diagnosis (n)		
Schizophrenia	6	6
Schizoaffective disorder	2	3
Affective psychosis with mood-incongruent features	1	2
Delusional disorder	2	2
Psychotic disorder NOS	6	4
Drug induced psychosis	3	3^a^
PANSS mean (range)		
Positive symptoms	18.7 (11–29)	10.4 (7–16)
Negative symptoms	16.6 (7–33)	9.1 (7–15)
General symptoms	36.4 (21–50)	24.4 (16–36)
Total symptoms	71.7 (46–112)	43.9 (30–63)
Number of Hospital ^b^ admissions mean (range)		2.5 (1–7)
Total time in hospital ^b^(mean weeks) (range)		33.8 (2–104)
QLF at 2 year follow-up^c^ mean (range)		
Evaluation of own health		3.6 (2–5)
Health in general		4.5 (2–6)
Physical condition		3.9 (2–6)
Emotional well-being		4.6 (3–7)
Life in general (repeated? question)		4.9 (3–7)

^a^At two year follow up these patients fullfilled DSM-IV citeria for 305.90 (Abuse)

^b^Patients’ satisfaction with different aspects of own life (Subjective Quality of Life) as measured by the “Delighted-terrible” scale

1 = Terrible - 7 = Delighted.

^c^patient from baseline to follow-up

The textual analysis resulted in four related themes, reflecting FEP patients’ experiences over the last 2 years. We refer to 14–19 participants as ‘most’, 8–13 as ‘many’, and to 3–7 as ‘some’ participants.

### Admission as a crucial stage

Most participants experienced admission in psychiatric wards to be a difficult and protracted process. It seems that the first meeting with the staff had a significant impact in giving a sense of powerlessness in some patients rather than compulsory admission itself.

*“I didn’t really have much that I could say”. “It was more like” “Now we are going to do this” and “Now we are going to do that”…. “So I had to do things their way”*

Compulsory admission was perceived as a particularly unpleasant experience, as it was seen as adding to the mental exhaustion caused by participants’ psychotic condition. Many patients retrospectively reported lack of insight (caused by their psychotic condition) at the time of admission, which caused a discrepancy between perceived symptom levels and symptom level assessment made by psychiatrists. This discrepancy was seen to cause resistance to hospitalization and reduced the willingness to adhere to AP treatment.

Also, many participants described a feeling of being left to their own devices during the initial phase of their stay. A perception of isolation (e.g. *I was sitting alone in the room at the ward*), being prohibited from exiting the ward, delay of medical assessment and lack of time with consultations during the first 2–3 days were all experiences highlighted as particularly challenging and described to reduce adherence to AP after discharge.

*“It was a nightmare, like you were imprisoned in the hospital, you can’t get out. It was quite a disgusting experience... ‘No, you can’t go outside today anyhow, there is not enough staff to do this. They could have talked to me about it as an equal human being in a way. Rather than saying ‘No’. They did not have a conversation with me. They let me just sit there in my room. How could they get to know me when they didn’t talk to me? I felt neglected. It was very frustrating. So I decided to discontinue my AP immediately after discharge”*

Having to remain at the hospital against their wishes also had a negative impact on their adherence to AP.

*“I was so tired of taking my AP because I’d been staying in psychiatry [a psychiatric ward] for almost one year and I used AP the entire time. I felt that I had no control over anything when I was there, so I took control over my medication when I was discharged and stopped taking them”*

On the other hand, some patients saw admission as necessary to counteract a severe psychotic condition. These patients were mostly satisfied with their stay and the care provided by the staff. They felt safe and warmly welcomed, perceiving staff members as supportive and open-minded. This gave a perception of being safeguarded, which made it easier to communicate their difficulties. Such an inclusive atmosphere was perceived to facilitate their endorsement of treatment as well as adherence to antipsychotic medication recommendations.

*“I’m very satisfied with the treatment I received. I got a lot of help. I felt very safe on the ward. I trusted NN (psychiatrist). She was fantastic … all the staff was actually like that…. I was difficult to deal with, I must admit, I wasn’t a very easy patient. I wasn’t violent, but I refused everything and initially I didn’t want to take any medicine. Nevertheless, they were able to convince me. I decided to use my AP for one or perhaps two years, then I thought I would be able to sustain myself without it (AP). I will adhere to my doctor’s recommendation”*

### Sufficient information at the right time

Most participants described getting insufficient information about effects and side effects of the AP they were initially allocated to. They saw this as particularly crucial in the early stages of the illness. For most participants, insufficient information evoked negative emotions such as frustration and anger, resulting in unfavorable alliances with staff which subsequently and reduced their adherence to AP.

*“The psychiatrist gave me rudimentary information about my medication. However, I remember that I went online and I looked for more information, I remember very well a situation when I just had increased the dose of my first AP. This led to suicidal thoughts and I proceeded to cut myself. Afterwards they informed me that this was a side effect of the medication. This hurt me a lot when I think about it. They had given me something wrong. I thought this is not my fault; it is the medicine’s fault and the doctor’s fault because he had not informed me about this side effect. I remember that I felt that I had received inadequate information. I made the choice not to use any sort of AP medication”*

Contrary to this, some of the participants felt that they got thorough information from professionals about AP effects and the most common side effects. These patients stated that they remembered the conversation with their psychiatrist about their AP at the acute stage. This type of information was linked to favorable adherence, reduced stress levels and helped patients cope with medication side effects.

*“I understood that they wanted me to try the medication. However, I think it is very important, that I should know about the side effects prior to use. I also had a medication counselling session where it was presented that the side effects were not strong at all, that these pills would have no serious consequences, and that it was fine to use them. So I decided to use them, and I’ve used them ever since”*

Most patients sought information about AP on their own, in most cases using online information sources. This approach was perceived to reduce stress, increase control and gave a perception of involvement and influence with regard to the process of initiating AP. In most cases, such approach increased adherence.

*“I’ve obtained a lot of information regarding the medication I used from the psychiatrist. I read the leaflet, I read a lot. Even though I didn’t understand all of it, I felt a sense of security from reading through it”*

Other recovered or non-recovered patients were considered by most participants as to be a particularly credible source of information about AP. First-hand experiences of other patients of using medication was seen as a decisive factor for trusting their advice. Also, seeing other patients’ physical and psychological reactions to medication affected participant adherence. If they perceived information about AP from staff as unclear, they often sought advice from peers. Other patients’ positive experiences with AP use could increase medical adherence and vice versa.

*“They gave me three options, one of which my friend had used in the past, and therefore, I didn’t want to use it, because I saw how it affects him… In addition, I heard that it caused a lot of weight gain and stuff like that, so I refused to use it.”*

*“The staff gave me information about my AP; however, I didn’t fully understand everything, so I attempted to talk with patients at the ward. I asked questions regarding how they reacted when they took their medicine… if it helped them sleep and what time they took it (e.g. before or after meal) and stuff like that. This made it much easier for me to learn about my medication”*

Information about the expected duration of AP use was unclear for most participants. Psychiatrists failed to present a timeframe in the beginning of the AP course as well as later. This was a source of concern for many, because they felt a need to know when they were going to be considered as recovered and able to cope without medication.

*“I tried to ask in the beginning for how long I would have to use this AP; however I had to give up, because I did not get any answers. They just said I should take it. When I asked for how long they just repeated their answer. They didn’t want to tell me, and that is actually very peculiar… It is fine that they were not sure, but it must be possible for them to tell me that the majority of psychotic patients use AP for one year, two years or half a year”*

Some patients got information about expected duration. This was perceived as having a positive influence on adherence.

*“I asked the psychiatrist what he assumed would be the duration of my using APs. Also I had a dialogue with my General Practitioner after discharge, and she stipulated minimum 6 months to one year. When I became stable she said “it’s time for discontinuing your AP”, so I used it for less than one year. This was my wish from the beginning; I told the physician that I would not like to use AP longer than necessary, because I usually don’t like to take any type of medicine”*

### A plea for shared in decision-making

Some patients reported being given the opportunity to decide which AP they could use. Some patients whom reported not being presented with a choice, retrospectively perceived this to be due to reduced or poor insight during the acute phase, recognizing this as a particular challenge for individuals with psychotic symptoms. However, lack of involvement reduced trust in staff as well as adherence in most cases.

*“They didn’t ask me what type of medication I wanted. I think that this was unfair of them. They said I could try it, and if I wasn’t satisfied with it, I could switch to another type of AP. They just prescribed it and they expected me to use it.”*

Most participants expressed a wish to be more involved in the choice of AP. They believed that this type of cooperation would increase ownership of their treatment. They wished to be treated as individuals and unique human beings, and not after a standard norm without distinctive features.

*“They didn’t offer me for example three medicines to choose from …. What are the advantages and disadvantages of each, and then I could have made more deliberate choice. I’ve only been offered one type and they said ‘this will help you’. Although they have competence, this doesn’t mean that they know it will help me. I’m not like just anyone else”*

Patients also reported a lack of involvement in decision-making when switching to a new AP. Although patients did not participate actively in decision-making, they were aware of the causes for switching, which were lack of efficacy and side effects. Perceived lack of involvement was tied to lack of adherence.

*“I was not involved in the decision making at all. I have always felt that they prescribed medicine to me without asking me, and when they switched to another one, they told me that first, we are going to decrease the dose of this one and that we then are going to start with a new one. I feel that I never played a part in the decision making concerning my own medication that I can’t decide what medicine I want to use…. This summer I’ve stopped taking any medication… and I think that I will not use medication any more. I don’t want to start with a new one either. I feel that starting up with a new medicine is a tiring experience; I notice it in my body.”*

### Attitude to antipsychotics echoes beneficial effects and illness insight

Most patients perceived AP as necessary to alleviate psychosis symptoms and subsequently normalize their mental health state.

Poor insight into their illness during the acute phase was perceived as making it harder for most patients to commit to any treatment, including AP treatment. However, as insight increased throughout the course, most patients reported increasingly recognizing the severity of their problems, and also that AP was a possible solution to reduce symptoms. The development of this attitude, resulting from increased insight, involved for many a more positive attitude towards AP and also thereby increased adherence.

*“When I was admitted to the psychiatric ward I was prescribed AP medication. I didn’t want to start using them. It took quite a long time before I realized that I was sick, and I noticed somehow that I didn’t have the opportunity to be like everyone else, whom can work and choose which school they want to go to… so I thought, yes I want to continue to take medicines, so I could function in the best way I can.”*

Some participants stated that they had an attitude of cautiousness and skepticism to all types of medicines, even before they became psychotic. For some, this made it difficult to listen to psychiatrists’ advice and adhere to recommendations. Nevertheless, the beneficial effects of AP use experienced by, that some of these participants’ experienced during the course, changed their view and made it easier to adhere.

*“I was very skeptical; I’ve always been like that, I’m against stuffing myself with any medication at all. However, after he (the doctor) explained it, it was a kind of…okay. If someone asks me, I will say that it was good; it is worth trying AP because it helps, and you get back on your feet faster. In my case, I recovered faster with the medication than without it, the symptoms disappeared. I’m very satisfied with it. It helped a lot with sleep; if I didn’t take them, then I couldn’t sleep.”*

For most patients, experiencing the desired effect of AP, even when accompanied with mild side-effects, was associated with favorable adherence. Also, a fear of a potential psychotic symptom relapse which might result from medication reduction or discontinuation was associated with positive attitudes to continued AP use.

*“The medication works very well and I was actually very satisfied with it. I have gained some weight, I have stopped smoking and then I have to work-out a bit more…. Now I use them every day, and I quickly notice that when I have forgotten to take them, I don’t feel so well… so I must take them … There has not been a single period where I have not used the medication… There won’t be a period like that... I know that if I don’t use the medication, then I will go back to being psychotic, and that is something that I don’t want… I would advise the others that have the same symptoms as I have to use the same medication that I used…don’t use another type”*

On the other hand, five out of the six participants whom experienced only problematic side effects in the absence of any positive effects chose to intentionally discontinue their AP. Certain side effects were of pivotal importance to them, e.g. weight gain and sedation.

*“Now, when I don’t use my medicine anymore I can truthfully say that I don’t notice any difference, the only thing that I notice is that I don’t experience any side effects. I usually struggle with eating and things like that, and when I began to gain weight, I stopped taking it immediately. If I will be admitted again and they offer me to use AP, then I wouldn’t accept it. It is my choice, I feel that the medicine doesn’t do me any good”*

Most participants reported having a period without AP during the last 2 years. Some participants didn’t use AP at the interview time, either because they decided to stop themselves or stopped in collaboration with their psychiatrist. Those who experienced deterioration after discontinuation of AP were careful not to cease taking their AP again. This was clearly reflected through their advice to other patients.

*“I have used AP since I became psychotic… if someone asked me, I would advise them to take the medication that their doctor recommends. We have to trust the doctor, especially if you are a first time patient; you have to take them if you need them. I advise that you certainly shouldn’t quit if you feel that the medications work as it is supposed to, because when you stop taking them, you will be worse just like before, so use them at least one year. It is easy to think that when you feel better and the symptoms disappear, you can just stop using AP because you think you don’t need them anymore. However, it is just because the medicine is working, as when you stop taking AP, you will be ill again. Many people make that mistake”*

Most of the participants whom were not using APs at the time of their interview, reported that they would be willing to start again with AP if they were to relapse and require re-hospitalized in the future.

*“I could use medicines again. I will take all the help that I can get”*

Patients, who were not willing to use AP again, stated that side effects were the main barrier. Lack of efficacy was also mentioned.

*“I will not use APs again. I’d probably try to find another method for healing. A method that I can do at home, that would perhaps help. This is because I have other problems, I struggle with food and I struggle with weight and stuff like that, so it would be a serious burden on me if I started gaining weight because of the AP. This would make me feel more sick and fed-up.”*

## Discussion

This study illuminates patients’ experiences of using AP and the possible incentives and barriers that influence adherence the first 2 years. The adherence issue has different dimensions which varies depending on both contextual and subjective factors. Each patient interpreted the world outside in their unique way and thus interacted with the clinicians differently. It seems that positive experiences of the hospital admission, sufficient and timely information, involvement in decision-making, as well as insight and the experience of beneficial effect of APs were seen to have a considerable impact on adherence to medication in our patient group.

Despite the fact that we did not have any key theme in the interview guide addressing admission experiences, all patients seemed to feel a need to talk about it, whether it was pleasant or not. It seems that the first contact with staff has a considerable impact on alliance and trust during the treatment process to follow, which in turn affects adherence to AP, especially after discharge. This supports findings from another study, that the reception of patients by medical staff has crucial impact on adherence, regardless of legal status of admission [42]. Also it might explain why patients in our study who felt that the staff violated their rights to freedom and authority to choose, chose to discontinue their medication after discharge as a way of trying to regain control over their own life, regardless of legal status when admitted.

Poor insight into illness alongside a perceived poor patient-staff alliance led to inadequate communication about medication. Patients mostly reported being not very receptive to any type of information during the acute stage, and this might be partly an explanation for their experience of not receiving enough information about their AP at this time. Moreover, the severity of illness and cognitive ability can be a determining element for assimilation of the information that had been given. Patients with psychiatric disorders often either forget or misunderstand information regarding their AP [43]. On the other hand, we can’t rule out that the staff did not see the need for complementary information at the acute stage of the illness when they considered that patients had poor insight. This may also justify the lack of involvement of patients in decision-making. Our data confirms previous findings showing that the shared decision-making perspective is different for psychiatrists and patients with schizophrenia [44]. A plea for involvement in decision-making may not merely mean the right to choose or change their antipsychotics. However, it may be extended to include authority, empowerment, control and even the right to not stay at the ward.

The feeling of being insufficiently informed about the use of AP led patients to seek information from Internet in most of the cases. A population survey has shown that 80% of all internet users have used the internet for information related to mental health [45]. This reveals a pressing need for professionals to develop a future system to handle a modern information exchange.

Poor insight, which is generally associated with negative attitudes to medication, seemed to affect adherence predominantly during the acute stage of illness. This is confirmed by existing research [26, 46]. On the other hand, when patients used AP for extended periods of time, other factors seemed to influence adherence. Many participants found the side effects of antipsychotics to be problematic and intolerable. This seems to support previous findings that not all patients can cope with medication side effects [47]. The use of AP medications entails a difficult trade-off between the benefit of alleviating psychotic symptoms and the risk of negative side effects [2].

## Limitations

Only two participants in our study got the opportunity to be active partners in shared decision-making, which makes it difficult for us to estimate its impact on adherence. Patients requested to be more involved in decisions concerning their medications, and this portrays the importance of this topic for them. Clinicians recognised insight as a significant barrier to shared decision making, assuming that patients in the acute stage are unable to make a decision [48]. The risk of nonadherence seems to increase when patients feel that they are excluded from treatment decisions. Decisions relating to medication are recognised as being some of the most important to patients and also one of the areas where they are most likely to disagree when they are not involved in the treatment process [44].

Six study eligible patients did not want to participate, and we do not know what opinions they night have offered. This can be considered as another limitation.

Strength of our study is that the first author was not a part of the treatment team for these patients. We believe that interviews conducted by a neutral person resulted in participants feeling able to talk more freely about their experiences.

Finally, five authors, from different professions (pharmacist, psychiatrist, psychiatric nurse, psychologist), performed analysis independently. We believe this increase the reliability and credibility of our results.

## Conclusion

Adherence to antipsychotic medication is a complicated phenomenon commensurate with complexity of human unique interaction and subjective interpretation of the world. Many elements have a prominent impact on it, and these factors may vary depending on patients’ experience during their illness journey. The patient-therapist relationship seems to be a crusial factor, and the initial contact with the patient is the key to the creation of an empowering alliance. This must be done with care and respect. Additionally, information about medication should be repeated once the person is out of the acutely psychotic stage of illness, as an interactive process throughout the course of treatment to strengthen the shared decision-making element of the treatment process.

## Abbreviations

DSM: Diagnostic and Statistical Manual of Mental Disorders
FEP: First-episode psychosis
L-QoLI: Lehman Quality of Life Interview
PANSS: Positive and Negative Syndrome Scale
SCID: Structured Clinical Interview for the DSM-IV Axis 1 Disorders
TIPS: Early intervention and treatment of psychosis
